# Exploring Unusual Cardiac Complications: Chorda Tendinea Rupture and Pulmonary Valve Vegetation in Infective Endocarditis—A Comprehensive Review

**DOI:** 10.7759/cureus.61401

**Published:** 2024-05-30

**Authors:** Nayakawadi Akhil, Amar Taksande, Revat J Meshram, Shailesh Wandile, Chaitanya Kumar Javvaji

**Affiliations:** 1 Paediatrics, Jawaharlal Nehru Medical College, Datta Meghe Institute of Higher Education and Research, Wardha, IND; 2 Pediatrics, Jawaharlal Nehru Medical College, Datta Meghe Institute of Higher Education and Research, Wardha, IND

**Keywords:** management, diagnosis, cardiac complications, pulmonary valve vegetation, chorda tendinea rupture, infective endocarditis

## Abstract

Infective endocarditis (IE) is a severe infection of the endocardium, frequently involving heart valves, and is associated with significant morbidity and mortality. At the same time, traditional complications of IE, such as valvular dysfunction and embolic events, are well-documented, and uncommon cardiac manifestations, such as chorda tendinea rupture and pulmonary valve vegetation, present unique diagnostic and management challenges. This comprehensive review explores the pathophysiology, clinical presentation, diagnostic strategies, and management approaches for IE's chorda tendinea rupture and pulmonary valve vegetation. Through a detailed examination of the literature and discussion of clinical scenarios, we highlight the importance of recognizing these rare complications and discuss the implications for clinical practice. Additionally, we identify knowledge gaps and propose areas for future research to enhance further our understanding and management of these unusual cardiac complications in IE. This review aims to provide clinicians with valuable insights to improve patient care and outcomes in the challenging setting of infective endocarditis.

## Introduction and background

Infective endocarditis (IE) is a severe and potentially life-threatening infection of the endocardium, typically involving the heart valves. It is characterized by the colonization and proliferation of microorganisms, usually bacteria, within the endocardial surface, leading to the formation of vegetation and potentially causing valvular dysfunction, embolic events, and systemic complications [1]. IE can affect individuals of all ages but is more prevalent in those with preexisting heart conditions, intravenous drug users, and individuals with prosthetic heart valves [2].

While the classic manifestations of IE, such as valvular dysfunction and embolic phenomena, are well-documented, there is a growing recognition of atypical or unusual cardiac complications associated with this condition [3]. These complications, including chorda tendinea rupture and pulmonary valve vegetation, present unique diagnosis, management, and prognostication challenges. Understanding these rare manifestations is crucial for clinicians to promptly recognize and appropriately manage them, thereby improving patient outcomes [4].

The purpose of this comprehensive review is to explore two uncommon cardiac complications of infective endocarditis: chorda tendinea rupture and pulmonary valve vegetation. We aim to provide insights into these rare entities through a detailed examination of the pathophysiology, clinical manifestations, diagnostic approaches, and management strategies. Furthermore, by discussing case studies and clinical scenarios, we intend to illustrate the real-world challenges encountered in diagnosing and treating these complications. Ultimately, this review seeks to contribute to the broader understanding of infective endocarditis and enhance clinical decision-making in managing patients with these complex cardiac conditions.

## Review

Pathophysiology of infective endocarditis

Microbial Pathogenesis

The microbial pathogenesis of infective endocarditis comprises several pivotal stages, including microbial adherence, binding to fibronectin, and sustained survival on the valvular surface. The process initiates with the preparation of the cardiac valve for bacterial adherence, precipitated by trauma-induced alterations in endothelial cells. These alterations render the surface conducive to colonization by circulating bacteria [5]. Subsequently, specific bacterial strains exhibit heightened adherence to the fibrin-platelet matrix, comprising platelets and fibrin, which accumulates at the injury site. The bacterial virulence factors facilitating adherence are multifaceted, with at least one, an extracellular polysaccharide (dextran), being identified [6]. Moreover, the survival of bacteria adhering to the vegetation surface appears intricate, necessitating inherent resistance to the bactericidal effects of complement and phagocytosis by white cells. Additionally, vegetation propagation entails the activation of the clotting cascade, culminating in the deposition and proliferation of a fibrin-platelet clot atop the rapidly expanding bacterial colonies [7]. The microbial pathogenesis of infective endocarditis encompasses a sequence of events commencing with the preparation of the cardiac valve for bacterial adherence, followed by adhesion and persistence of bacteria on the valvular surface, and ultimately, the proliferation of infected vegetation via activation of the clotting cascade [7].

Host Factors

**Figure 1:**
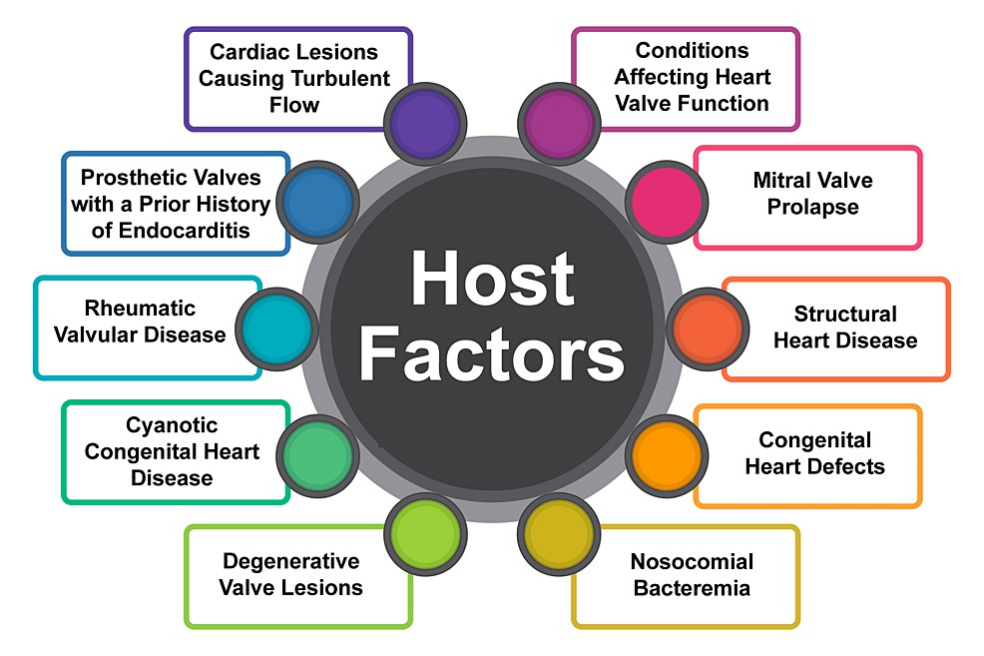
Host factors for infective endocarditis

Host factors that elevate the susceptibility to infective endocarditis encompass a spectrum of conditions, notably including cardiac lesions inducing turbulent flow, prosthetic valves with a prior history of endocarditis, rheumatic valvular disease, cyanotic congenital heart disease, degenerative valve lesions, and mitral valve prolapse (Figure 1). Additionally, intravenous drug use and nosocomial bacteremia are emerging as pivotal factors among patients predisposed to endocarditis [8]. Furthermore, other predisposing factors for infective endocarditis entail structural heart disease, congenital heart defects, rheumatic valvular disease, and conditions compromising heart valve function, thereby augmenting the risk of complications and heart failure [9].

Valve Anatomy and Vulnerability

The heart has four valves responsible for directing the flow of blood: the aortic, mitral, tricuspid, and pulmonary valves [10]. Each valve's distinct anatomy influences its disease susceptibility [10,11]. The mitral valve comprises two leaflets, the anterior and posterior, supported by chordae tendineae that attach to papillary muscles [10]. In contrast, the tricuspid valve has three leaflets, while the aortic and pulmonary valves feature three cusps [10,11]. Notably, the left-sided valves (mitral and aortic) exhibit slightly greater thickness than their right-side counterparts [10]. The unique anatomical characteristics of each valve predispose them to specific pathologies. For instance, the mitral valve's two-leaflet structure renders it susceptible to prolapse and potential chordae tendineae rupture [12]. Conversely, the aortic valve is prone to stenosis, particularly among elderly individuals, owing to calcification along the valve leaflets [13]. Despite all valves serving the everyday function of regulating blood flow, their anatomical distinctions contribute to their susceptibility to disease. A profound comprehension of valve anatomy is imperative for promptly recognizing and effectively managing valvular heart disorders.

Traditional complications of infective endocarditis

Valvular Dysfunction

Valvular dysfunction denotes the impairment of heart valves' capacity to open and close effectively, disrupting blood flow regulation within the heart. This dysfunction can arise from various conditions, including infective endocarditis, which has the potential to inflict damage upon the heart valves and precipitate regurgitation (leakage) or stenosis (narrowing) of the valves [14,15]. Within the context of infective endocarditis, valvular dysfunction may ensue due to the direct impact of the infection on the heart valves. The infection can instigate the formation of vegetation on the valves, tissue degradation, and the emergence of abscesses, all of which can compromise the valves' standard functionality [14,15]. Valvular dysfunction in infective endocarditis may manifest as regurgitation, characterized by incomplete valve closure, allowing blood to flow backward, or stenosis, marked by valve narrowing, impeding blood flow through the heart chambers [14,15]. Such dysfunction can profoundly affect heart function, resulting in fatigue, dyspnea, and cardiac murmurs. Left untreated, it can progress to heart failure and other grave complications. Timely detection of valvular dysfunction in infective endocarditis is paramount for instigating appropriate treatment, which may encompass antibiotic therapy to manage the infection and, in severe instances, surgical intervention to rectify or replace the compromised heart valves. Vigilant monitoring and effective management of valvular dysfunction in infective endocarditis are imperative for averting further complications and optimizing patient outcomes [16].

Embolic Events

Embolic events are a notable complication of infective endocarditis (IE) in many cases. These events entail the formation of emboli that traverse the bloodstream, potentially occluding blood vessels and precipitating severe consequences such as stroke, myocardial infarction, or organ failure [17-19]. The risk of embolic events in IE is influenced by various factors, including the dimensions of vegetation on the heart valves, specific bacteria such as Staphylococcus aureus, and abscesses or other cardiac complications [17-19]. Notably, vegetation size emerges as a robust predictor of embolic events, with more extensive vegetation (>10 mm) associated with a heightened risk of embolization [18,19]. Early identification of these risk factors assumes paramount importance in the effective management of IE, as it enables clinicians to pinpoint patients who may derive benefit from early surgical intervention to avert further embolization. Indeed, research indicates that prompt surgical intervention can significantly diminish the incidence of embolic events in IE patients [18,19]. Although the incidence of embolic events in IE exhibits variability across studies, it is generally reported to range between 20% and 40% of cases [20]. This underscores the significance of vigilant patient monitoring for indications of embolization and the implementation of strategies to prevent or manage these complications adeptly.

Heart Failure

In a particular study, 78% of 40 consecutive endocarditis patients exhibited congestive heart failure (CHF) at the time of diagnosis [21]. Another investigation revealed that 61% of patients developed CHF during their hospitalization [22]. Notably, patients afflicted with endocarditis who manifest heart failure tend to experience inferior outcomes compared to their counterparts devoid of CHF [22]. Heart failure constitutes a significant contributor to morbidity and mortality in these instances [23]. In the context of endocarditis, heart failure often results from severe valve regurgitation resulting from valve destruction induced by the infection [21,23]. Acute valve dysfunction precipitates abrupt volume overload and subsequent pump failure. Risk factors for heart failure in endocarditis encompass pre-existing valve disease, prosthetic valves, and virulent organisms such as Staphylococcus aureus [21,23]. Advanced age also serves as a risk factor. Notably, symptoms of heart failure such as dyspnea, oedema, and fatigue may serve as the presenting signs of endocarditis in certain patients [23]. Progression to cardiogenic shock can transpire rapidly. The treatment approach entails administering antibiotics to manage the infection, diuretics and afterload reduction agents for heart failure, and frequently, urgent valve surgery to excise infected tissue and reinstate valve competence [21,23].

Chorda tendinea rupture: mechanisms and clinical implications

Anatomy and Function of Chorda Tendinea

The chordae tendineae, often called the heartstrings, comprise inelastic cords of fibrous connective tissue that establish connections between the papillary muscles and the tricuspid and mitral valves within the heart. These integral structures serve a critical role in averting the prolapse of valve leaflets during ventricular systole by tensing and retaining the flaps closed, thereby forestalling the backward flow of blood into the atria [24]. Functionally, during atrial systole, when blood transitions from the atria to the ventricles, the chordae tendineae remain relaxed as the atrioventricular valves open. Conversely, during ventricular systole, the heightened blood pressure in both chambers prompts the atrioventricular valves to close concurrently, prompting the chordae tendineae to tense and forestall valve leaflet prolapse, thereby preserving closure [25]. From a clinical perspective, ruptured chordae tendineae can precipitate severe mitral insufficiency, inducing noteworthy hemodynamic alterations and mandating expeditious intervention to avert complications such as heart failure. An adept comprehension of the anatomy and function of chordae tendineae is imperative for accurately diagnosing and effectively managing conditions associated with these vital structures [25].

Etiology of Chorda Tendinea Rupture in Infective Endocarditis

The aetiology of chordae tendineae rupture in infective endocarditis encompasses many factors, with infective endocarditis as a prominent causative factor. This condition can precipitate acute mitral regurgitation through mechanisms such as leaflet perforation and alterations in the mitral valve annulus secondary to abscess formation [26]. Primary rupture and its association with various connective tissue disorders are also implicated in chordae tendineae rupture [26]. A retrospective study at the Sheba Medical Center, Tel-Hashomer, Israel, shed light on the leading etiologies of ruptured chordae tendineae in hospitalized patients, identifying infective endocarditis and primary rupture as predominant factors. The study underscored that patients with primary rupture of the chordae tendineae tended to be older than those with endocarditis, with the posterior mitral valve cusp being more frequently involved. Furthermore, the presence of mitral valve prolapse was frequently observed among patients with infective endocarditis, suggesting a potential predisposition to chordae tendineae rupture in this subgroup [26]. Hence, the aetiology of chordae tendineae rupture in infective endocarditis involves factors such as direct damage to the valve structures by the infective process, age-related changes, and potential underlying structural abnormalities like mitral valve prolapse. Early recognition and appropriate management of infective endocarditis assume paramount importance in averting complications such as chordae tendineae rupture and mitigating adverse outcomes in affected patients [27].

Clinical Manifestations and Diagnostic Challenges

The clinical manifestations and diagnostic intricacies associated with chordae tendineae rupture present significant challenges in diagnosis and management. Clinical presentations often encompass symptoms indicative of acute heart failure, including dyspnea, chest pain, and signs of hemodynamic instability such as cardiogenic shock. Additionally, patients may manifest murmurs, abnormal heart sounds, and echocardiographic findings suggestive of severe mitral regurgitation [28-30]. Diagnostic challenges ensue from the diverse clinical presentations of chordae tendineae rupture, which can mimic other cardiac conditions like cardiac amyloidosis or acute coronary syndromes. The spectrum of differential diagnoses may encompass valvular heart disease, infective endocarditis, and other structural heart abnormalities. Accurate diagnosis requires a comprehensive evaluation integrating clinical assessment, imaging modalities such as echocardiography, and occasionally invasive procedures like cardiac catheterization or tissue biopsy to confirm the underlying pathology [28-30]. The complexity inherent in diagnosing chordae tendineae rupture is further compounded by the imperative to differentiate between partial and complete ruptures, ascertain the extent of mitral regurgitation, and determine the optimal surgical approach for repair or replacement of the affected valve. Multidisciplinary collaboration among cardiologists, cardiac surgeons, and imaging specialists is paramount in navigating these diagnostic challenges and devising individualized treatment strategies to optimize patient outcomes [29,30].

Management Strategies and Prognosis

Managing mitral regurgitation resulting from ruptured chordae tendineae entails a multifaceted approach encompassing medical and surgical interventions. In cases of acute rupture, initial treatment involves medical support, including intravenous administration of inotropes, diuretics, and vasodilators to stabilize the patient [31]. However, surgical intervention is often imperative to address the underlying pathology and restore valve function. Surgical options typically involve mitral valve repair, which may entail leaflet plication without resection or plication after wedge resection of the unsupported leaflet [32]. In instances where repair is not feasible or ineffective, mechanical valve replacement may be considered [31]. The prognosis for patients with mitral regurgitation following chordae tendineae rupture varies based on factors such as underlying comorbidities, the extent of the rupture, and the timeliness of intervention. Studies have indicated favourable survival rates, with a 92% survival rate at five years and 73% at ten years for patients undergoing repair of ruptured chordae tendineae of the mitral valve [32]. Nonetheless, surgical repair carries inherent risks, including operative mortality and postoperative complications such as neurological issues [31]. Hence, achieving optimal management necessitates a balanced consideration of the patient's clinical condition and the chosen surgical approach. A multidisciplinary approach, integrating medical stabilization and surgical intervention, is fundamental to achieving favourable outcomes in patients with mitral regurgitation due to ruptured chordae tendineae. While surgical outcomes have demonstrated promising long-term survival rates, meticulously evaluating each patient's circumstances and carefully selecting the appropriate surgical strategy is crucial to maximizing prognosis and mitigating complications [33].

Pulmonary valve vegetation: rarity, characteristics, and management

Overview of Pulmonary Valve Involvement in Infective Endocarditis

Pulmonary valve infective endocarditis (PVIE) is a rare manifestation, comprising only about 1.5-2% of all cases of infective endocarditis (IE) [34,35]. Its occurrence is more prevalent among individuals with congenital heart disease or those with predisposing factors such as intravenous drug abuse or central venous catheters [36,37]. Patients presenting with PVIE may exhibit symptoms such as prolonged febrile illness, right heart failure, septic pulmonary emboli, and pulmonary regurgitation [36]. Diagnosis can pose challenges due to the rarity of the condition, but suspicion should be raised in febrile patients with congenital disabilities, bacteremia, or central venous lines [36]. Trans-thoracic echocardiography is a cornerstone in diagnosing PVIE, revealing characteristic features like vegetations, valve thickening, and mobile masses [36,37]. Additionally, computed tomography imaging can aid in identifying complications such as pulmonary emboli or aneurysms [36]. Management of PVIE typically involves a combination of prolonged antibiotic therapy and often surgical intervention, particularly in cases featuring large, mobile vegetations causing hemodynamic compromise or recurrent emboli [34,37]. Surgical approaches may encompass vegetation excision, valve repair, or replacement, contingent upon the extent of tissue damage [34]. PVIE can carry a high mortality rate, especially in instances of delayed diagnosis, fulminant septicemia, or multi-organ failure [36]. Hence, early detection and prompt intervention are paramount to enhancing outcomes and averting complications. In conclusion, pulmonary valve involvement in infective endocarditis is a rare yet potentially life-threatening condition necessitating a vigilant approach, including a high index of suspicion, multi-modality imaging for diagnosis, and a multidisciplinary management strategy comprising medical therapy and surgical intervention to optimize patient outcomes [34-37].

Characteristics and Imaging Findings of Pulmonary Valve Vegetations

Pulmonary valve vegetations represent a rare occurrence, affecting approximately 2% of patients diagnosed with infective endocarditis. These vegetations, often sizable and highly mobile, can give rise to severe complications such as pulmonary embolism and acute respiratory failure [38]. Imaging techniques, notably echocardiography and cardiovascular magnetic resonance (CMR) imaging, play pivotal roles in diagnosing and evaluating pulmonary valve vegetation characteristics. Echocardiography, encompassing two-dimensional and Doppler echocardiography, is indispensable for identifying the underlying mechanisms of pulmonary valve vegetation. It enables visualization of features such as flail or dysplastic cusps, restricted cusp mobility, or mal-coaptation of the pulmonary valve. Furthermore, echocardiography aids in assessing the severity of pulmonary regurgitation by examining the width and duration of the regurgitant jet, providing insights into the condition's severity [39]. Conversely, CMR imaging furnishes detailed insights into the morphology and hemodynamics of pulmonary valve vegetations. CMR facilitates visualization of valve leaflets, right ventricular enlargement, and other structural abnormalities associated with pulmonary valve vegetations. Utilizing two-dimensional cine images acquired through steady-state free procession imaging and phase-contrast imaging, CMR allows for evaluating both antegrade and retrograde flows in the main artery below the pulmonary artery bifurcation [40]. The characterization and imaging assessment of pulmonary valve vegetations relies on echocardiography and CMR imaging to visualize the vegetations, evaluate their mobility, and assess associated complications such as pulmonary embolism. These imaging modalities are indispensable for effectively diagnosing and managing patients with pulmonary valve vegetation [41].

Diagnostic Approaches and Challenges

In clinical manifestation, infective endocarditis (IE) manifests with a broad spectrum of symptoms, encompassing fever, fatigue, new heart murmurs, and embolic phenomena. However, these symptoms lack specificity and may overlap with manifestations of other medical conditions, posing challenges in diagnosis [42,43]. Microbiological diagnosis of IE predominantly relies on blood cultures, the cornerstone for identifying causative organisms. Nonetheless, in instances of culture-negative endocarditis, additional diagnostic approaches such as molecular techniques and prolonged blood culture incubation may be necessary to ascertain the responsible pathogens [43,44]. Imaging modalities, notably echocardiography in transthoracic and transesophageal forms, assume pivotal roles in IE diagnosis by facilitating the detection of vegetations, valvular abnormalities, and complications like abscess formation. Emerging as a valuable adjunct to echocardiography, multislice computed tomography (CT) is gaining recognition for its utility in diagnosing IE [44,45]. Challenges in IE research persist despite its significant clinical impact, primarily attributable to deficiencies in research infrastructure and funding. This dearth contributes to a scarcity of randomized controlled trials essential for informing clinical practice, perpetuating unresolved controversies such as the optimal timing of surgery and the role of antibiotic prophylaxis [42]. Regarding treatment implications, the prompt and accurate diagnosis of IE is imperative for initiating appropriate antibiotic therapy and considering surgical intervention as warranted. Delayed diagnosis can precipitate serious complications and escalate mortality rates [44,45].

Treatment Modalities and Outcomes

Managing infective endocarditis (IE) typically entails a combination of antimicrobial therapy and, frequently, surgical intervention [46-48]. The selection of treatment modality is contingent upon various factors, including the causative pathogen, the severity of the infection, the presence of complications, and the patient's overall health status [46-48]. Initial empiric antimicrobial therapy is often broad-spectrum, guided by patient characteristics, prior antibiotic exposures, and epidemiological considerations [48]. Subsequently, upon pathogen identification and receipt of susceptibility results, the antimicrobial regimen is customized accordingly [48]. The duration of antimicrobial therapy varies depending on the type of IE (native valve vs. prosthetic valve) and the symptoms preceding diagnosis [48]. For native valve endocarditis attributable to penicillin-susceptible enterococci, a four-week course of therapy is recommended for patients presenting with symptoms <3 months, a six-week course is advocated for those with symptoms persisting ≥3 months or prosthetic valve endocarditis [48]. Surgical intervention is often indispensable for patients with IE to excise infected tissue, debride abscesses, repair or replace damaged valves, and eradicate the infection [46,47]. Indications for surgery encompass heart failure, uncontrolled infection, prevention of embolism, and prosthetic valve dysfunction [47]. The outcomes of surgical management for IE generally demonstrate favourable results, with an overall hospital survival rate of approximately 90% [47]. Notably, patients with prosthetic valve endocarditis exhibit higher 30-day mortality rates compared to those with native valve endocarditis, although long-term survival rates are comparable [47]. IE attributable to Staphylococcus aureus is associated with markedly elevated mortality rates compared to other pathogens [47].

Future directions and research opportunities

Advancements in Imaging Techniques

Positron emission tomography (PET), specifically 18F-fluorodeoxyglucose PET combined with computed tomography (18F-FDG PET/CT), has demonstrated notable efficacy in diagnosing prosthetic valve endocarditis and cardiac device-related infections, boasting high sensitivity (87%) and specificity (94%) [49,50]. This imaging modality highlights areas of heightened glucose metabolism corresponding to active inflammation, offering valuable insights into disease activity [49]. Single-photon emission computed tomography (SPECT), employing 99mTc-HMPAO-SPECT/CT with technetium-99m-hexamethylpropyleneamine oxime-labeled autologous leukocytes, delves into ongoing infection at a molecular level, aiding in the determination of optimal therapeutic strategies in infective endocarditis (IE) [51]. Molecular imaging is continuously evolving, with emerging modalities like PET and SPECT furnishing additional diagnostic value by assessing patients at risk of IE and facilitating accurate diagnosis [51]. These modalities provide crucial insights into ongoing infection dynamics and are pivotal in guiding treatment decisions [51]. Adopting a multimodality approach is advocated for a comprehensive assessment of IE, with a combination of imaging techniques such as echocardiography, PET/CT, and SPECT/CT recommended [50,52,53]. This integrated approach enhances diagnostic accuracy, particularly in complex cases, and represents a significant stride in advancing the diagnosis of IE, notably benefiting high-risk patients with prosthetic valves or intracardiac devices. Consequently, incorporating PET/CT and SPECT/CT alongside echocardiography is now endorsed in current guidelines for diagnosing IE [50,52,53].

Emerging Therapeutic Approaches

Research indicates a growing interest in exploring non-antibiotic, antimicrobial strategies in managing infective endocarditis (IE), with a particular focus on anti-thrombotic interventions and hyperbaric oxygen therapy aimed at targeting biofilm formation on heart valve vegetations [54]. These novel approaches seek to tackle the challenging nature of IE by disrupting biofilm structures associated with the disease, potentially leading to enhanced treatment outcomes [54]. In adjunctive therapies for IE, studies have delved into the potential benefits of combining additional treatments with standard antibiotic therapy. For instance, investigations have highlighted the thrombin inhibitor dabigatran as a promising adjunctive treatment strategy, mainly when administered alongside antibiotic (gentamicin) therapy in vivo, showcasing the potential for improved treatment efficacy [54]. Future research endeavours in IE should prioritize the development of innovative diagnostic methods, mainly focusing on enhancing the detection of blood culture-negative infective endocarditis (BCNIE) [45]. Novel approaches such as targeted metagenomics on whole blood and plasma and 16S rRNA gene-targeted next-generation sequencing hold promise for enabling more accurate pathogen identification in IE cases [45]. Recognizing the complexity of IE management, a clinically integrated multidisciplinary approach is advocated, highlighting the significance of collaborative efforts among various medical specialities to ensure comprehensive care for patients with IE [55]. This collaborative approach can help optimize treatment strategies, foster interdisciplinary communication, and improve patient outcomes.

## Conclusions

In conclusion, this comprehensive review has delved into the intricate complexities surrounding two uncommon cardiac complications of infective endocarditis: chorda tendinea rupture and pulmonary valve vegetation. Significant insights have emerged through a meticulous examination of their pathophysiology, clinical presentations, diagnostic challenges, and management strategies. Chorda tendinea rupture presents as an acute valvular regurgitation necessitating emergent surgical intervention, while pulmonary valve vegetation, although rare, demands vigilant monitoring due to its potential for embolic complications. These findings hold profound implications for clinical practice, emphasizing the importance of maintaining a high index of suspicion for these complications, employing a multidisciplinary approach for prompt diagnosis, and tailoring treatment strategies to individual patient needs. However, notable knowledge gaps persist amidst these advancements, urging a call to action for further research endeavors. Prospective studies are needed to elucidate risk factors, prognostic indicators, and optimal management approaches, ultimately enhancing our ability to identify and manage these rare cardiac complications effectively. By addressing these research priorities, we can advance the field of infective endocarditis and ultimately improve patient outcomes in the future.
